# A network meta-analysis assessing the effectiveness of various radical and conservative surgical approaches regarding recurrence in treating solid/multicystic ameloblastomas

**DOI:** 10.1038/s41598-023-32190-7

**Published:** 2023-05-25

**Authors:** Faqi Nurdiansyah Hendra, Marco N. Helder, Muhammad Ruslin, Ellen M. Van Cann, Tymour Forouzanfar

**Affiliations:** 1 Department of Oral and Maxillofacial Surgery/ Oral Pathology, Amsterdam UMC and Academic Centre for Dentistry Amsterdam (ACTA), Vrije Universiteit Amsterdam, Cancer Center Amsterdam, Amsterdam, The Netherlands; 2 Department of Anatomy, Faculty of Medicine, Hasanuddin University, Makassar, Indonesia; 3 Department of Oral and Maxillofacial Surgery, Faculty of Dentistry, Hasanuddin University, Makassar, Indonesia; 4 Department of Head and Neck Surgical Oncology, UMC Utrecht Cancer Center, University Medical Center Utrecht, Utrecht, The Netherlands

**Keywords:** Cancer, Oncology

## Abstract

Multiple treatment approaches have been undertaken to reduce the incidence of recurrence in solid/multicystic ameloblastoma (SMA), both conservative and radical. A network meta-analysis (NMA) was conducted to assess and compare the effectiveness of these various treatment approaches concurrently. This study was reported based on the Preferred Reporting Items for Systematic Reviews for Network Meta-Analysis (PRISMA-NMA) statement. PubMed (MEDLINE), ScienceDirect, Scopus, and Web of Science were searched until August 10, 2021. The NMA was conducted using the STATA program. Of 1153 records identified in the search, seven observational studies with 180 patients were included. Six different treatment approaches were identified. Segmental resection ranked highest for reducing the recurrence rate with the highest SUCRA score (77.7), followed by curettage with cryotherapy (66.9) and marginal resection (49.3). Network inconsistencies and publication bias appeared to be absent. According to the Confidence in Network Meta-Analysis (CINeMa) method, the evidence's certainty was low for all comparisons due to imprecision and within-study bias. In conclusion, this study is the first NMA in the field of ameloblastoma. Segmental resection seemed to be the most effective treatment approach for minimizing recurrence in SMA patients. Nevertheless, weak certainty of evidence makes that the results must be regarded with caution.

## Introduction

Ameloblastoma is a rare benign odontogenic tumor of epithelial origin that makes up around 10% of all tumors in the jaws. Despite being considered benign, ameloblastoma has a locally invasive development. Around 70% of cases progress to malignancy, and up to 2% of cases spread to other organs^1,2^. Ameloblastoma is classified into three types according to the 2017 World Health Organization (WHO) classification of benign epithelial odontogenic tumors: ameloblastoma (solid/multicystic/conventional ameloblastoma), unicystic ameloblastoma, and peripheral ameloblastoma^3^.

Solid/multicystic ameloblastoma (SMA) is the most prevalent type and appears more aggressive than other types based on recurrence rates^4,5^. SMAs mostly occur in the posterior mandible of patients aged 30–40 years, without gender or ethnicity preference^6,7^. The most common histopathological pattern of SMA is follicular, followed by plexiform and other rare patterns: acanthomatous, desmoplastic, basaloid, and granular^8^.

The main treatment is surgery, which may be classified into two modalities: radical and conservative. Radical surgical approaches include *en bloc* or marginal and segmental resections with wide (1–2 cm) safety bone margins. Conservative surgical approaches consist of enucleation, curettage, and marsupialization, followed by additional treatment, such as peripheral ostectomy, cryotherapy, or Carnoy’s solution^9–11^. Our previous systematic review and meta-analysis discovered that the radical approach is the treatment of choice for SMA patients due to a reduced recurrence rate^5^. However, it usually requires reconstructive procedures and greatly affects the patient's quality of life after surgery. Contrarily, conservative therapy can minimize operating time while maintaining the patient's quality of life, however, associated with a high incidence of recurrence^12,13^.

Besides our previous study^5^, there have also been several systematic reviews and meta-analyses that compare radical treatment versus conservative treatment in SMA patients^6,7,14–16^. Still, no studies have compared several (more than two) approaches of each modality simultaneously and specifically due to the limitations of conventional meta-analysis methods that can only compare a pair of interventions. In recent years, a popular and increasingly recognized technique has been developed to overcome this problem, which is an advanced form of paired meta-analysis called network meta-analysis (NMA)^17^.

NMA is the best method of compiling evidence and selecting the most valuable treatment from many studies that compare numerous interventions. It can estimate direct and indirect comparative efficacies and provide a ranking among all interventions. Moreover, integrating both direct and indirect evidence can produce more precise estimates^17–20^. Hence, by implementing this new method in the present study, we aim to evaluate the efficacy of various radical and conservative surgical approaches in terms of recurrence rate for the treatment of SMA patients.

## Material and methods

### Protocol registration

This NMA was conducted according to PRISMA for Network Meta-analyses (PRISMA-NMA) Guidelines^21^. The protocol was registered on PROSPERO (ID: CRD42021271539).

## Research question and eligibility criteria

We planned to investigate and answer the following research question: “Which radical and conservative treatment approach results in lower recurrence rates in SMA patients?”. The following eligibility criteria were used: Participants (P): Human patients with primary SMA. Interventions (I): Radical surgical approaches (segmental resection, marginal resection) and conservative surgical approaches (enucleation, curettage, the combination between them, and with or without adjuvant therapy). Comparators (C): All interventions (surgical approaches) will be compared with each other. Outcome (O): Recurrence rate. Study design (S): Randomized/non-randomized controlled trials and observational studies that compared at least two interventions (surgical approaches). Case reports and reviews were excluded.

The exclusion criteria were: recurrent SMA treatment; former marsupialization or decompression, irradiation, or prior therapy at a different facility than the one where the research was conducted; unicystic, peripheral, and metastasizing ameloblastomas; a follow-up duration is not stated; non-English languages studies; in vitro and animal studies, reviews, case reports, and case series with fewer than 10 participants.

### Searches and information sources

PubMed (MEDLINE), Scopus, ScienceDirect, and Web of Science databases were used to search the articles published up to August 2021 (date of the last search: August 10, 2021), utilizing a combination of search phrases: “ameloblastoma”, “radical OR conservative”, and “recurrence OR relapse”. Furthermore, manual searches of the articles’ reference list were conducted to locate more relevant publications not found in the databases. The details of the search strategy are presented in Supplementary Table 1.

### Study selection, data selection process, and data items

Two independent reviewers (F.N.H. & M.N.H.) conducted the article selection process blinded to each other. Disagreements among the reviewers were settled through discussion. A third reviewer (T.F.) was consulted if necessary. The search histories were saved and exported to the reference management program (Mendeley Desktop, Version 1.19.8). Duplicate records were removed afterwards.

In the first stage of screening process, titles and abstracts from remaining records were screened for possible inclusion. In the second stage, the full text of the articles was screened for final inclusion. Studies with no full-text available or data that was incomplete or ambiguous were omitted.

Author, publication year, study country or region, study design, demographic data of participants, tumor and histopathologic type, treatment modality, recurrences linked to the treatment method, and post-operative follow-up period were extracted from full-text articles using a data extraction form and stored in Microsoft Excel program for each study. We also checked for information regarding adjuvant therapy given to primary SMA patients in all included studies, but none provided such information.

### Interventions of interest

The interventions of interest were the first and primary surgical treatments of SMA patients, divided into radical and conservative approaches. The radical approach consists of segmental resection, marginal resection, hemimandibulectomy, or total mandibulectomy. The conservative approach includes enucleation, enucleation plus curettage, enucleation with Carnoy’s solution, enucleation plus cryotherapy, enucleation plus peripheral ostectomy, curettage, curettage plus cryotherapy, other or a combination of the previous.

### Outcome of interest

The primary outcome of interest was a recurrence, defined as ameloblastoma coming back at the original site or a distant location.

### Quality assessment

Risk of bias in non-randomized studies-of exposure (ROBINS-E)^22^ tool was used to assess the risk of bias within studies. This tool sets seven domains of bias: confounding, measurement of the exposure, selection of participants, post-exposure interventions, missing data, measurement of the outcome, and selection of the reported result. The assessment was graded as low risk, medium risk (some concerns), or high risk. For the overall risk of bias results, the studies were classified as low risk if all domains are at low risk except for concerns in the confounding domain, as medium risk if at least one domain is at some concerns but no domains are at high risk, and as high risk if at least one domain is at high risk of bias. The results were displayed as the risk of bias graph and summary using RevMan 5.4 program (Review Manager. The Cochrane Collaboration, 2020).

To assess the certainty of evidence in network meta-analysis, the Confidence in Network Meta-Analysis (CINeMA) web tool was employed, which evaluated the following aspects: within-study bias, indirectness, imprecision, heterogeneity, incoherence, and reporting bias. For each comparison, the confidence level was rated as high, moderate, low, or very low^23–25^.

### Strategy for data synthesis

A network meta-analysis was conducted using mvmeta and network packages in Stata program (Stata SE. Version 16.0. StataCorp LLC. College Station, TX, USA)^26^. We estimated the odds ratios (ORs) with 95% confidence intervals (CIs) for each comparison and displayed the results in the interval plot or network league table. The geometry of the treatment network was shown visually via the network map or diagram.

Inconsistency was assessed through two stages. The first is to test overall inconsistency globally using the design-by-treatment interaction model, calculated using the Wald test. The second is to use the loop-specific approach, which evaluates inconsistencies separately in each closed loop of network interventions. The inconsistency factor (IF) is assessed in each loop as the absolute difference between direct and indirect estimations for one of the loop's comparisons. A 95% CI and a *z* test for IF were also calculated. Loops with statistically significant inconsistency are those in which the lower CI limit of the IF does not reach zero. If inconsistencies are detected, sensitivity and meta-regression analyses are used to explore potential inconsistency causes^20,26–28^.

We evaluated the potential publication bias using a net funnel plot^29^. The surface under the cumulative ranking (SUCRA) curve was used to rank the treatment approach and plotted the results in rankogram to identify which treatment approach is the best^30^.

## Results

### Study selection and characteristics

**Figure 1:**
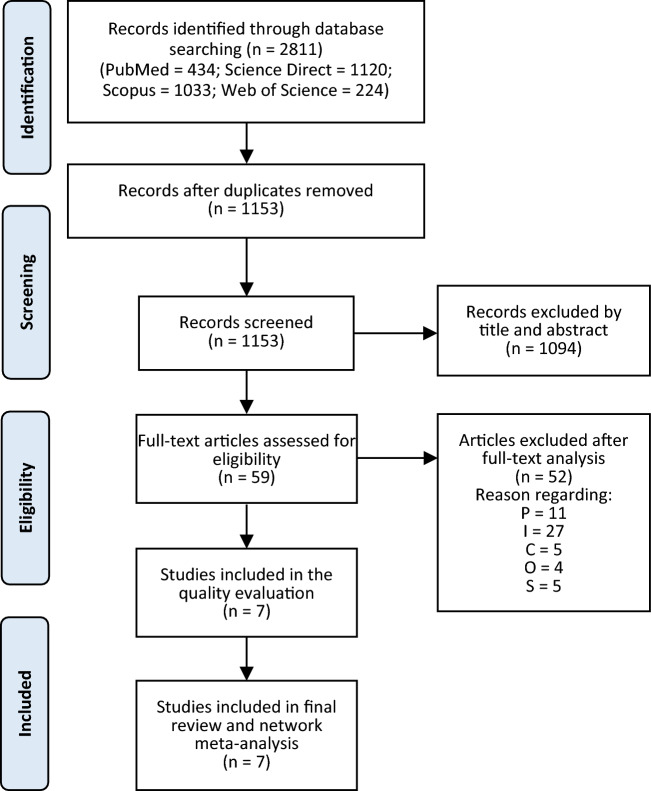
The study selection process diagram.

A total of 2811 records were found in multiple databases throughout the search. We screened 1153 records by titles and abstracts after eliminating duplicates. A total of 59 articles were considered for full-text screening, with 23 of them being eliminated later. The reasons for article exclusion are listed in Supplementary Table 2. Subsequently, seven studies^31–37^ with 180 SMA patients and 38 recurrences from several countries in Europe, Asia, North America, and South America were included in the quality evaluation and incorporated in the review and network meta-analysis. Figure 1 depicts the study selection procedure. All studies included were retrospective cohort studies. The mean age of patients was approximately 36.8 years. The follicular pattern was the most common histopathological subtype (37%), followed by the plexiform pattern (34.7%). There were several surgical approaches to radical treatment, such as segmental resection (SR) and marginal resection (MR); as well as conservative treatment options such enucleation, enucleation and curettage (ENCU), enucleation with the Carnoy's solution (ECS), and curettage with cryotherapy (CCR). Table 1 summarizes the characteristics of the studies that were included.

**Table 1:** The characteristics of the studies that were included.

Study & country	Number of SMA	Age of patients	Treatment approach	Recurrence	Histopathological subtype (recurrence)	Follow-up period
Chapelle et al. 2004^31^ Netherlands	14	Median: 43 years (17–77)	Segmental resection = 2 Marginal resection = 2 Enucleation + Carnoy's solution = 4 Enucleation = 6	0 0 1 3	Follicular = 7 (2) Plexiform = 2 (0) Follicular + Plexiform = 5 (2)	Mean: 8.8 years (1–20 years)
Curi et al. 1997^32^ Brazil	36	Mean: 31 years	Marginal resection = 5 Curettage + Cryotherapy = 31	2 9	NA	Mean: 62 months (14 months–18 years)
Hasegawa et al. 2013^33^ Japan	17^a^	Mean: 38.8 years	Enucleation + Curettage = 7 Enucleation = 10	2 4	Follicular (3) Plexiform (2) Desmoplastic (1)	8–130 months
Hong et al. 2007^34^ South Korea	51^b^	Mean: 34.5 years	Segmental resection = 19 Marginal resection = 32	1 5	Follicular = 15 (3) Plexiform = 21 (0) Acanthomatous = 9 (2) Granular cell = 5 (1) Desmoplastic = 1 (0)	More than 1 year
Junquera et al. 2003^35^ Spain	12	Mean: 44.5 years	Segmental resection = 5 Marginal resection = 2 Enucleation + Curettage = 5	1 1 2	Follicular = 5 (1) Plexiform = 4 (1) Acanthomatous = 1 (1) Granular cell = 1 (1) Desmoplastic = 1 (0)	2–23 years
Nakamura et al. 2002^36^ Japan	40^a^	Mean: 34.1 years	Segmental resection = 25 Marginal resection = 4 Enucleation + Curettage = 11	3 0 2	Follicular = 13 (2) Plexiform = 16 (1) Follicular + Plexiform = 8 (2) Desmoplastic = 3 (0)	More than 5 years
Petrovic et al. 2018^37^ USA	10	Median: 61.5 years (19–81)	Segmental resection = 9 Marginal resection = 1	2 0	Follicular = 7 (1) Plexiform = 1 (0) Acanthomatous = 1 (1) Granular cell = 1 (0)	Mean: 69.2 months (1–196 months)
Total	180		180	38		

*SMA* solid/multicystic ameloblastoma, *NA* Not available.

^a^Treatment with marsupialization was excluded. ^b^Conservative treatment was excluded because the approach was not specified.

### Risk of bias in individual studies

**Figure 2:**
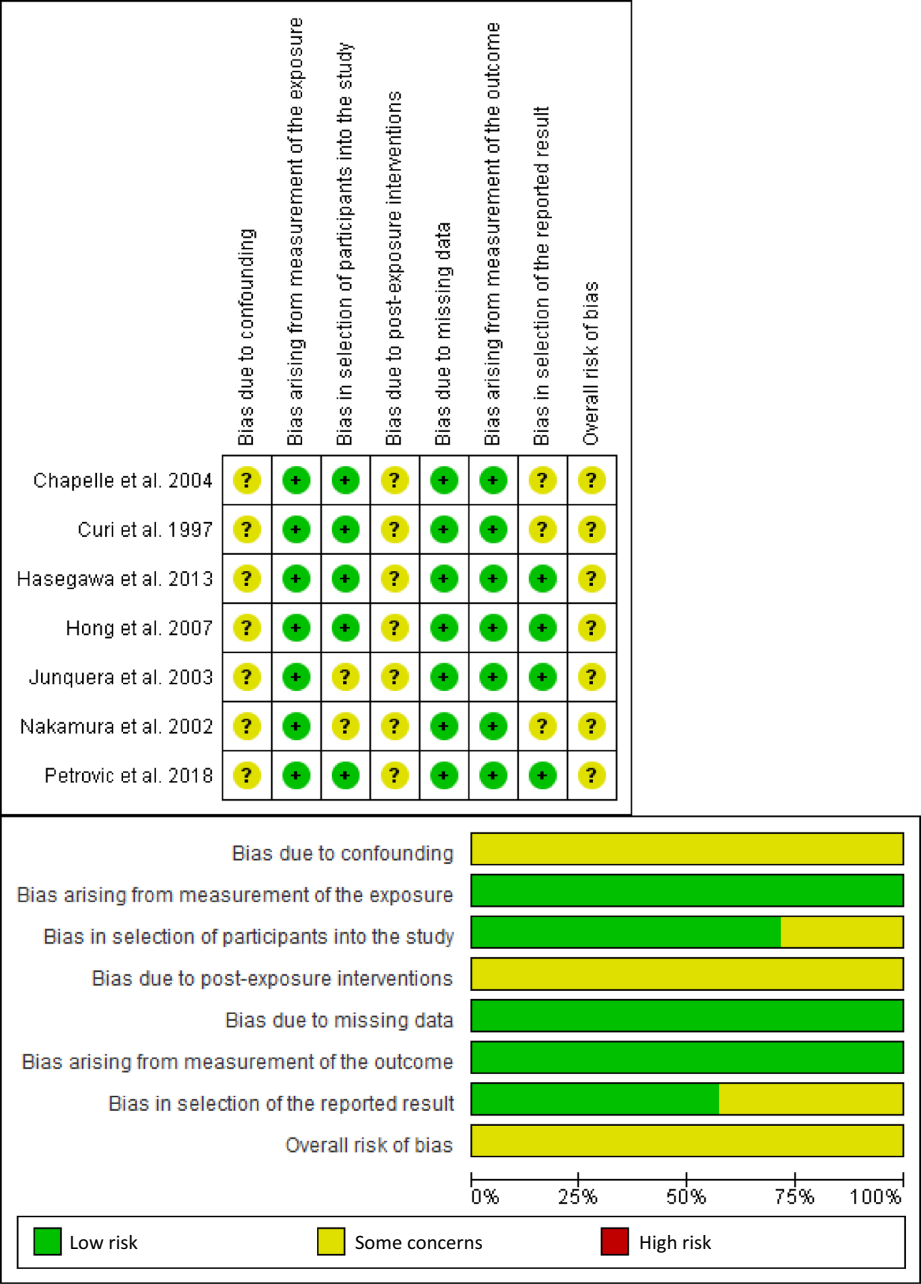
Risk of bias graph & risk of bias summary of individual studies.

For the overall risk of bias, all the studies had a medium risk of bias. Regarding the domain assessment, all the studies had some concerns in confounding and post-exposure intervention domains. They had a low risk of bias at missing data and measurement of the exposure and outcome domains. Two studies had some concerns about selecting participants, and three had concerns about selecting the reported result. Figure 2 shows the risk of bias graph and summary of the studies that were included.

### Network geometry and inconsistency

**Figure 3:**
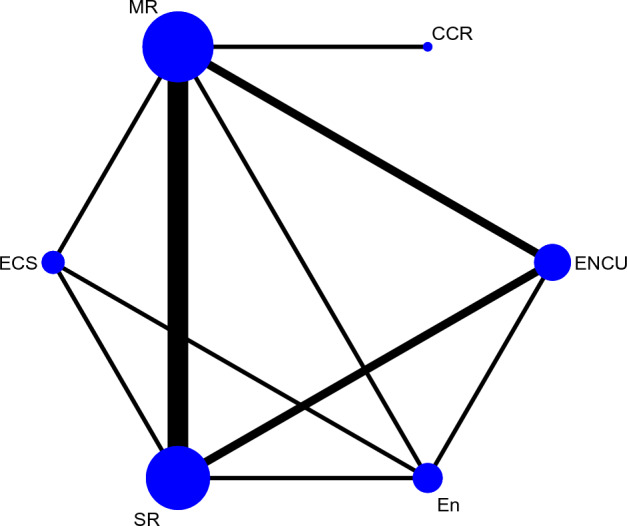
Network map of treatment approach comparisons. The size of the nodes describes the total sample size of treatment approaches. The thickness of the lines correlates to the number of studies that are compared. CCR = Curettage + Cryotherapy, ECS = Enucleation + Carnoy’s solution, En = Enucleation, ENCU = Enucleation + Curettage, MR—Marginal resection, SR—Segmental resection.

Ten direct pairwise comparisons of treatment approaches were available in the network map. The most common comparators were MR, SR, and enucleation, respectively. The number of studies in each treatment comparison were SR versus MR (5), MR versus ENCU (2), SR versus ENCU (2), MR versus CCR (1), MR versus ECS (1), enucleation versus ENCU (1), SR versus enucleation (1), enucleation versus ECS (1), SR versus ECS (1), and MR versus enucleation (1). Furthermore, 15 indirect pairwise comparisons were made. The network map of treatment approach comparisons is shown in Fig. 3. For inconsistency in the network, five closed loops were identified, including the treatment approaches of ECS, enucleation, ENCU, MR, and SR. These loops had acceptable IF values, and the overall* p*-value for network inconsistency was 0.96, which meant no violation of the consistency assumption for direct and indirect estimates (Supplementary Table 3).

### Network meta-analysis outcome

The network league of treatment approach comparisons is presented in Table 2. Compared to enucleation only, the odds ratio (OR) of recurrence rate for SR, CCR, MR, ENCU, and ECS were 0.22 (95% confidence interval (CI), 0.03 – 1.43), 0.24 (95% CI, 0.01 – 3.98), 0.39 (95% CI, 0.05 – 2.95), 0.47 (95% CI, 0.09 – 2.59), and 0.48 (95% CI, 0.05 – 5.05) respectively. Compared to ECS, OR of SR, CCR, MR, and ENCU were 0.45 (95% CI, 0.03 – 6.31), 0.50 (95% CI, 0.02 – 13.96), 0.81 (95% CI, 0.05 – 12.12), and 0.97 (95% CI, 0.07 – 13.67) consecutively. Compared to ENCU, OR of SR, CCR, and MR were 0.46 (95% CI, 0.12 – 1.81), 0.51 (95% CI, 0.04 – 6.60), and 0.83 (95% CI, 0.16 – 4.37) respectively. Compared to MR, OR of SR and CCR were 0.56 (95% CI, 0.14 – 2.20) and 0.61 (95% CI, 0.09 – 4.31). Comparison of SR with CCR had an OR of 0.91 (95% CI, 0.08 – 9.86).

**Table 2:** Network league of treatment approach comparisons for recurrence outcome using Odds Ratio (OR) to measure the effect size.

SR					
0.91 (0.08, 9.86)	CCR				
0.56 (0.14, 2.20)	0.61 (0.09, 4.31)	MR			
0.46 (0.12, 1.81)	0.51 (0.04, 6.60)	0.83 (0.16, 4.37)	ENCU		
0.45 (0.03, 6.31)	0.50 (0.02, 13.96)	0.81 (0.05, 12.12)	0.97 (0.07, 13.67)	ECS	
0.22 (0.03, 1.43)	0.24 (0.01, 3.98)	0.39 (0.05, 2.95)	0.47 (0.09, 2.59)	0.48 (0.05, 5.05)	En

*MR* marginal resection, *SR* segmental resection.

*CCR = Curettage + Cryotherapy, ECS = Enucleation + Carnoy’s solution, En = Enucleation, ENCU = Enucleation + Curettage.

**Figure 4:**
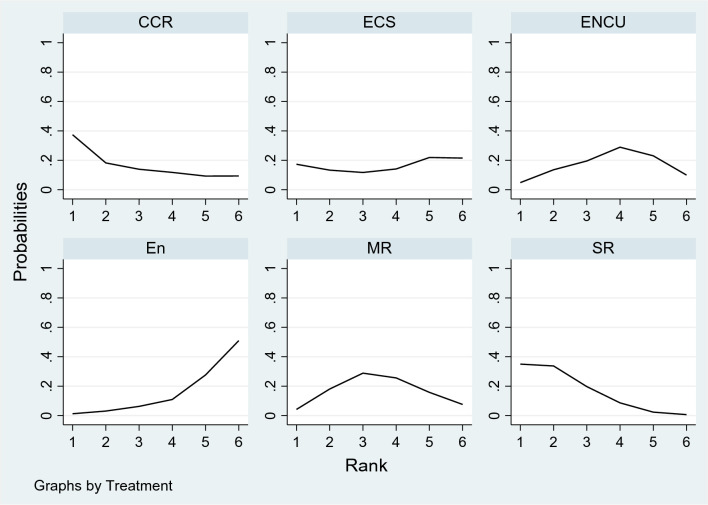
Rankograms for the ameloblastoma treatments network showing the probability of every treatment being in a particular order. CCR = Curettage + Cryotherapy, ECS = Enucleation + Carnoy’s solution, En = Enucleation, ENCU = Enucleation + Curettage, MR—Marginal resection, SR—Segmental resection.

Based on SUCRA values, SR had the highest mean rank (2.1) for lowering the recurrence rate (SUCRA score 77.7) in the rankogram, followed by CCR (SUCRA score 66.9) and MR (SUCRA score 49.3). The SUCRA value and the rankogram for the ameloblastoma treatment approach network are shown in Table 3 and Fig. 4. The relative ranking of treatments using the multidimensional scaling (MDS) approach showed the same results that segmental resection was the best treatment approach to reduce the incidence of recurrence (Supplementary Figure 1).

**Table 3:** The SUCRA value of each ameloblastoma treatment approach with regard to the recurrence rate.

Treatment	SUCRA	PrBest	Mean rank
En	17.3	1.2	5.1
CCR	66.9	37.4	2.7
ECS	45.1	17.3	3.7
ENCU	43.7	4.9	3.8
MR	49.3	4.2	3.5
SR	77.7	35.0	2.1

*MR* marginal resection, *SR* segmental resection.

*CCR = Curettage + Cryotherapy, ECS = Enucleation + Carnoy’s solution, En = Enucleation, ENCU = Enucleation + Curettage.

### Publication bias and evidence's certainty

Publication bias or risk of bias across studies was unlikely to be detected, as indicated by the symmetrical funnel plot (Supplementary Figure 2). The certainty of the evidence was low for all comparisons due to imprecision and within-study bias. The imprecision occurs because the confidence intervals of all pairwise treatment comparisons include a value of one, which indicates no difference in effect between the two treatments. Supplementary Table 4 shows the confidence ratings for the treatment approach comparisons.

## Discussion

To our knowledge, this is the first NMA of ameloblastoma treatment. Our prior systematic review found a higher recurrence rate in SMA patients with the conservative treatment approach than with the radical approach^5^. Nevertheless, even within conservative and radical treatments, approaches vary widely. By using the NMA method, we wanted to analyse in more detail what the best treatment modality of those various approaches (four types of conservative and two types of radical treatment) was in reducing the recurrence rate of SMA. Of 1153 records identified in the search, seven observational studies with 180 patients were included. We found that based on the network league and rankogram results, segmental resection ranked highest for reducing the recurrence rate with the highest SUCRA score (77.7), followed by curettage with cryotherapy (66.9) and marginal resection (49.3). Enucleation appeared the worst to reduce the recurrence rate in SMA patients. However, the confidence interval of all treatment approach comparisons includes one, which means the results are not statistically significant. This, coupled with the low certainty of the evidence, makes the results obtained need to be interpreted with caution.

SR is a radical surgical approach with discontinuity of the jawbone. This approach is usually accompanied by immediate or delayed bone repair with tissue grafts and prosthesis rehabilitation to aid speech and mastication in post-operative patients^10,34,38,39^. The results of this present study are in line with several reviews that state that SR is the preferred treatment for preventing SMA recurrence^40–42^. The meta-analysis of Almeida et al.^6^ also showed that SR appeared to be better than MR at reducing recurrence rates for SMA patients. However, the results were not statistically significant owing to a scarcity of samples or studies.

Considering the results of the SUCRA scores and the relative ranking of treatments, the best treatment approach after SR is CCR, a combination of conservative surgical modalities. Cryotherapy is an additional treatment approach that uses freezing to eradicate remaining tumor cells by inducing cellular necrosis while preserving the inorganic osseous structure^43–45^. These results indicate that the combination of conservative treatments still has the potential to be used in SMA patients, especially for those in which treatments are not possible or have contraindications for getting radical treatment. Examples are elderly patients who are physically weak and vulnerable^46,47^, or pediatric patients who require consideration of several other factors such as the occurrence of dysfunction, deformity, impaired growth of the face, as well as psychological effects after surgery^48,49^. These results also show that combining several conservative treatment approaches is still better at reducing the recurrence rate than using a single conservative approach. This is consistent with several reviews which state that using a single conservative approach such as simple enucleation is not recommended for SMA patients. Although this procedure has a low morbidity rate and provides outstanding aesthetic and functional outcomes, its drawback is the high recurrence rate (60–80%)^42,50^.

The high rate of ameloblastoma recurrence after treatment is still a major issue today. This recurrence rate is correlated to several factors, including the type of genetic mutation, the ameloblastoma variant based on its histopathology, and the treatment method^12,51,52^. SMA, the most common and aggressive variant of ameloblastoma, was significantly correlated with recurrence, especially for the follicular pattern with acanthomatous and basal cell alterations^53^.

This NMA includes seven studies that matched the eligibility criteria, all of which were retrospective cohort studies. The rare incidence of ameloblastoma (with a 0.9 per million annual incidence rate)^54^ with slow-growing characteristics accompanied by the recommendation for a post-treatment follow-up period of more than five years, makes it difficult for researchers to conduct prospective studies or randomized clinical trials (RCT) on the treatment of ameloblastoma. Not surprisingly, until now, there has not been a single RCT in this field.

Several limitations were found in this present study. Firstly, our review includes only a small number of studies with relatively small sample sizes yielding many analyses having low confidence in their results. Secondly, only retrospective cohort studies were included and analyzed in this study, the design of which provides a low degree of scientific evidence based on the Oxford Centre for Evidence-Based Medicine’s standards^55,56^. Furthermore, we could not account for any confounding factors within studies that may have affected the outcome with that design. Lastly, only English-language literature was searched.

## Conclusions

Our network meta-analysis showed SR seemed to be the best treatment approach for reducing recurrence in SMA patients. If radical treatment is not feasible for the patient, conservative treatment with multiple approaches, such as CCR, is indicated. However, the certainty of confidence in the results is still considered weak. Therefore, further studies with optimal methodological standards and long post-operative follow-up duration are needed to strengthen the evidence.

## Supplementary Information

Supplementary Information.

## Data Availability

The authors confirm that all data generated or analysed during this study are included in this published article and its supplementary information files. Raw data supporting this study's findings are available from the corresponding author on reasonable request.
