# Supplementary material for: A network meta-analysis assessing the effectiveness of various radical and conservative surgical approaches regarding recurrence in treating solid/multicystic ameloblastomas
Source: Sci Rep. 2023 May 25;13:8445. doi: 10.1038/s41598-023-32190-7 (PMC10213029; doi:10.1038/s41598-023-32190-7)
Supplement: Supplementary file 1 — Supplementary Information. [file 41598_2023_32190_MOESM1_ESM.pdf]

A network meta-analysis assessing the effectiveness of various radical and conservative surgical approaches regarding recurrence in treating solid/multicystic ameloblastomas:

Supplementary information files

Faqi Nurdiansyah Hendra [1,2,*], Marco N. Helder [1], Muhammad Ruslin [3], Ellen M. Van Cann [1,4], Tymour Forouzanfar [1]

[1] Department of Oral and Maxillofacial Surgery/Pathology, Amsterdam UMC and Academic Centre for Dentistry Amsterdam (ACTA), Vrije Universiteit Amsterdam, Cancer Center Amsterdam, Amsterdam, the Netherlands
[2] Department of Anatomy, Faculty of Medicine, Hasanuddin University, Makassar, Indonesia
[3] Department of Oral and Maxillofacial Surgery, Faculty of Dentistry, Hasanuddin University, Makassar, Indonesia
[4] Department of Head and Neck Surgical Oncology, UMC Utrecht Cancer Center, University Medical Center Utrecht, Utrecht, the Netherlands

***** Corresponding author: Faqi Nurdiansyah Hendra

List of contents

Title	page
Supplementary Table 1. Search strategy and search results details.	2
Supplementary Table 2. The reasons for the excluded studies.	3
Supplementary Table 3. Network inconsistency.	4
Supplementary Table 4. Confidence assessments in network meta-analysis of treatment approach comparisons.	5
Supplementary Table 5. SUCRA values for the ameloblastoma treatments network. (A) estimated probabilities; (B) predictive probabilities.	6
Supplementary Table 6. PRISMA 2020 checklist.	7
Supplementary Table 7. PRISMA network meta-analysis checklist.	9
Supplementary Figure 1. Relative ranking of treatments for the ameloblastoma network based on the multidimensional scaling (MDS) approach.	13
Supplementary Figure 2. Comparison-adjusted funnel plot of the ameloblastoma treatments network.	14
Supplementary Figure 3. Interval plot of treatment approach comparisons for recurrence outcome using Odds Ratio (OR) to measure the effect size.	15
Supplementary Figure 4. Network forest plots of treatment approach comparisons.	16
Supplementary Figure 5. Cumulative probability curves for the ameloblastoma treatments network show that each treatment's estimated and predictive probabilities are up to a specific rank.	17

Supplementary Table 1. Search strategy and search results details.

Database	Search Query	Date	Number of Results
PubMed (Medline)	ameloblastoma AND (radical OR conservative OR resection) AND (recurrence OR relapse) ("ameloblastoma"[MeSH Terms] OR "ameloblastoma"[All Fields] OR "ameloblastomas"[All Fields]) AND ("radical"[All Fields] OR "radical s"[All Fields] OR "radicals"[All Fields] OR ("conservancies"[All Fields] OR "conservancy"[All Fields] OR "conservancy s"[All Fields] OR "conservation"[All Fields] OR "conservational"[All Fields] OR "conservations"[All Fields] OR "conservative"[All Fields] OR "conservatively"[All Fields] OR "conservatives"[All Fields] OR "conserve"[All Fields] OR "conserved"[All Fields] OR "conserves"[All Fields] OR "conserving"[All Fields]) OR ("resect"[All Fields] OR "resectability"[All Fields] OR "resectable"[All Fields] OR "resectates"[All Fields] OR "resected"[All Fields] OR "resecting"[All Fields] OR "resection"[All Fields] OR "resectional"[All Fields] OR "resectioned"[All Fields] OR "resectioning"[All Fields] OR "resections"[All Fields] OR "resective"[All Fields] OR "resects"[All Fields])) AND ("recurrance"[All Fields] OR "recurrence"[MeSH Terms] OR "recurrence"[All Fields] OR "recurrences"[All Fields] OR "recurrencies"[All Fields] OR "recurrency"[All Fields] OR "recurrent"[All Fields] OR "recurrently"[All Fields] OR "recurrents"[All Fields] OR ("recurrence"[MeSH Terms] OR "recurrence"[All Fields] OR "relapse"[All Fields] OR "relapses"[All Fields] OR "relapsing"[All Fields] OR "relapsed"[All Fields] OR "relapser"[All Fields] OR "relapsers"[All Fields]))	10-Aug-21	434
ScienceDirect	ameloblastoma AND (radical OR conservative OR resection) AND (recurrence OR relapse)	10-Aug-21	1120
Scopus	ameloblastoma AND (radical OR conservative OR resection) AND (recurrence OR relapse)	10-Aug-21	1033
Web of Science	ameloblastoma AND (radical OR conservative OR resection) AND (recurrence OR relapse)	10-Aug-21	224
		TOTAL	2811

Supplementary Table 2. The reasons for the excluded studies.

Reason for exclusion	Articles excluded
No data about the histopathological type	Saraiya 2020; Adeel et al. 2018; Hammarfjord et al. 2013; Chaine et al. 2009; Chana et al. 2004; Arotiba et al. 1997; Olaitan & Adekeye 1996; Olaitan et al. 1993; Muller & Slootweg 1985; Holland & Mellor 1991; Adekeye 1980
Failure to differentiate histopathological type regarding treatment used	Goh et al. 2021; Hresko et al. 2021; Okechi et al. 2020; Menon et al. 2019; Au et al. 2019; Laborde et al. 2017; Milman et al. 2016; Franca et al. 2012; Li et al. 2012; Dandriyal et al. 2011; Rastogi et al. 2010; Escande et al. 2009; Sammartino et al. 2007; Adebayo et al. 2005; Hatada et al. 2001; Sampson & Pogrel 1999; Chidzonga et al. 1996; Pinsolle et al. 1995; Ueno et al. 1989; Sehdev et al. 1974
Not specifying the treatment approach	Goh et al. 2021; Hresko et al. 2021; Singh et al. 2015; Ghandhi et al. 2006;
Only one type of treatment used	Haq et al. 2016; Ooi et al. 2014; Carneiro et al. 2014; Bianchi et al. 2013; Bataineh 2000; Vedtofte et al. 1978
Recurrence is unclear regarding the type of treatment	Vongsa et al. 2013; Zhang et al. 2010; Molla et al. 1991
Recurrence is unclear regarding the treatment of the primary tumor	Hertog et al. 2012; Fregnani et al. 2010
Possibility of duplicate data	Hertog et al. 2012; Olaitan et al. 1993
Case reports or fewer than 10 cases	Singh et al. 2014; Andrade et al. 2013; Carneiro et al. 2014; Huang et al. 2007; Zwahlen & Gratz 2002;
Follow-up is not specified or unclear	Okechi et al. 2020; Giraddi et al. 2018; Vongsa et al. 2013; Franca et al. 2012; Gunawardhana et al. 2010; Vayvada et al. 2006; Arotiba et al. 1997; Chidzonga et al. 1996; Sehdev et al. 1974

Supplementary Table 3. Network inconsistency.

chi2(3) = 0.33
Prob > chi2 = 0.9547

Loop	IF	seIF	z_value	p_value	CI_95	Loop_Heterog_tau2
C-D-E	0.858	2.340	0.367	0.714	(0.00,5.45)	0.000
D-E-F	0.687	2.550	0.269	0.788	(0.00,5.68)	0.000
B-E-F	0.687	2.695	0.255	0.799	(0.00,5.97)	0.000
C-D-F	0.443	2.164	0.204	0.838	(0.00,4.68)	0.000
C-E-F	0.304	1.661	0.183	0.855	(0.00,3.56)	0.000
B-D-E	.	.	.	.		0.000
B-D-F	.	.	.	.		0.000

Notes: B = Enucleation + Carnoy's solution, C = Enucleation, D = Enucleation + Curettage, E =Marginal resection, F = Segmental resection.

Supplementary Table 4. Confidence assessments in network meta-analysis of treatment approach comparisons.

Comparison	Number of studies	Within-study bias	Reporting bias	Indirectness	Imprecision	Heterogeneity	Incoherence	Confidence rating	Reason(s) for downgrading
CCR:MR	1	Some concerns	Low risk	No concerns	Major concerns	No concerns	No concerns	Low	["Within-study bias", "Imprecision"]
ECS:En	1	Some concerns	Low risk	No concerns	Major concerns	No concerns	No concerns	Low	["Within-study bias", "Imprecision"]
ECS:MR	1	Some concerns	Low risk	No concerns	Major concerns	No concerns	No concerns	Low	["Within-study bias", "Imprecision"]
ECS:SR	1	Some concerns	Low risk	No concerns	Major concerns	No concerns	No concerns	Low	["Within-study bias", "Imprecision"]
En:ENCU	1	Some concerns	Low risk	No concerns	Major concerns	No concerns	No concerns	Low	["Within-study bias", "Imprecision"]
ENCU:MR	2	Some concerns	Low risk	No concerns	Major concerns	No concerns	No concerns	Low	["Within-study bias", "Imprecision"]
ENCU:SR	2	Some concerns	Low risk	No concerns	Major concerns	No concerns	No concerns	Low	["Within-study bias", "Imprecision"]
En:MR	1	Some concerns	Low risk	No concerns	Major concerns	No concerns	No concerns	Low	["Within-study bias", "Imprecision"]
En:SR	1	Some concerns	Low risk	No concerns	Major concerns	No concerns	No concerns	Low	["Within-study bias", "Imprecision"]
MR:SR	5	Some concerns	Low risk	No concerns	Major concerns	No concerns	No concerns	Low	["Within-study bias", "Imprecision"]
CCR:ECS	0	Some concerns	Low risk	No concerns	Major concerns	No concerns	No concerns	Low	["Within-study bias", "Imprecision"]
CCR:ENCU	0	Some concerns	Low risk	No concerns	Major concerns	No concerns	No concerns	Low	["Within-study bias", "Imprecision"]
CCR:En	0	Some concerns	Low risk	No concerns	Major concerns	No concerns	No concerns	Low	["Within-study bias", "Imprecision"]
CCR:SR	0	Some concerns	Low risk	No concerns	Major concerns	No concerns	No concerns	Low	["Within-study bias", "Imprecision"]
ECS:ENCU	0	Some concerns	Low risk	No concerns	Major concerns	No concerns	No concerns	Low	["Within-study bias", "Imprecision"]

Notes: CCR = Curettage + Cryotherapy, ECS = Enucleation + Carnoy's solution, En = Enucleation, ENCU = Enucleation + Curettage, MR = Marginal resection, SR = Segmental resection.

Supplementary Table 5. SUCRA values for the ameloblastoma treatments network. (A) estimated probabilities; (B) predictive probabilities.

A

Treatment	SUCRA	PrBest	MeanRank
En	17.3	1.2	5.1
CCR	66.9	37.4	2.7
ECS	45.1	17.3	3.7
ENCU	43.7	4.9	3.8
MR	49.3	4.2	3.5
SR	77.7	35.0	2.1

B

Treatment	SUCRA	PrBest	MeanRank
En	17.6	1.0	5.1
CCR	67.0	37.4	2.7
ECS	45.7	18.7	3.7
ENCU	43.0	4.8	3.8
MR	49.4	4.2	3.5
SR	77.2	34.0	2.1

Notes: CCR = Curettage + Cryotherapy, ECS = Enucleation + Carnoy's solution, En = Enucleation, ENCU = Enucleation + Curettage, MR = Marginal resection, SR = Segmental resection.

Supplementary Table 6. PRISMA 2020 checklist.

Section and Topic	Item #	Checklist item	Location where item is reported (page)
**TITLE**			
Title	1	Identify the report as a systematic review.	1
**ABSTRACT**			
Abstract	2	See the PRISMA 2020 for Abstracts checklist.	1
**INTRODUCTION**			
Rationale	3	Describe the rationale for the review in the context of existing knowledge.	2
Objectives	4	Provide an explicit statement of the objective(s) or question(s) the review addresses.	2
**METHODS**			
Eligibility criteria	5	Specify the inclusion and exclusion criteria for the review and how studies were grouped for the syntheses.	3
Information sources	6	Specify all databases, registers, websites, organisations, reference lists and other sources searched or consulted to identify studies. Specify the date when each source was last searched or consulted.	3
Search strategy	7	Present the full search strategies for all databases, registers and websites, including any filters and limits used.	3
Selection process	8	Specify the methods used to decide whether a study met the inclusion criteria of the review, including how many reviewers screened each record and each report retrieved, whether they worked independently, and if applicable, details of automation tools used in the process.	3
Data collection process	9	Specify the methods used to collect data from reports, including how many reviewers collected data from each report, whether they worked independently, any processes for obtaining or confirming data from study investigators, and if applicable, details of automation tools used in the process.	3
Data items	10a	List and define all outcomes for which data were sought. Specify whether all results that were compatible with each outcome domain in each study were sought (e.g. for all measures, time points, analyses), and if not, the methods used to decide which results to collect.	3
	10b	List and define all other variables for which data were sought (e.g. participant and intervention characteristics, funding sources). Describe any assumptions made about any missing or unclear information.	3
Study risk of bias assessment	11	Specify the methods used to assess risk of bias in the included studies, including details of the tool(s) used, how many reviewers assessed each study and whether they worked independently, and if applicable, details of automation tools used in the process.	4
Effect measures	12	Specify for each outcome the effect measure(s) (e.g. risk ratio, mean difference) used in the synthesis or presentation of results.	4
Synthesis methods	13a	Describe the processes used to decide which studies were eligible for each synthesis (e.g. tabulating the study intervention characteristics and comparing against the planned groups for each synthesis (item #5)).	4
	13b	Describe any methods required to prepare the data for presentation or synthesis, such as handling of missing summary statistics, or data conversions.	4
	13c	Describe any methods used to tabulate or visually display results of individual studies and syntheses.	4
	13d	Describe any methods used to synthesize results and provide a rationale for the choice(s). If meta-analysis was performed, describe the model(s), method(s) to identify the presence and extent of statistical heterogeneity, and software package(s) used.	4
	13e	Describe any methods used to explore possible causes of heterogeneity among study results (e.g. subgroup analysis, meta-regression).	Not Applicable
	13f	Describe any sensitivity analyses conducted to assess robustness of the synthesized results.	Not Applicable

Section and Topic	Item #	Checklist item	Location where item is reported (page)
Reporting bias assessment	14	Describe any methods used to assess risk of bias due to missing results in a synthesis (arising from reporting biases).	4
Certainty assessment	15	Describe any methods used to assess certainty (or confidence) in the body of evidence for an outcome.	4
**RESULTS**			
Study selection	16a	Describe the results of the search and selection process, from the number of records identified in the search to the number of studies included in the review, ideally using a flow diagram.	5 & 13
	16b	Cite studies that might appear to meet the inclusion criteria, but which were excluded, and explain why they were excluded.	5
Study characteristics	17	Cite each included study and present its characteristics.	5 & 11
Risk of bias in studies	18	Present assessments of risk of bias for each included study.	5 & 14
Results of individual studies	19	For all outcomes, present, for each study: (a) summary statistics for each group (where appropriate) and (b) an effect estimate and its precision (e.g. confidence/credible interval), ideally using structured tables or plots.	5 & 6
Results of syntheses	20a	For each synthesis, briefly summarise the characteristics and risk of bias among contributing studies.	5
	20b	Present results of all statistical syntheses conducted. If meta-analysis was done, present for each the summary estimate and its precision (e.g. confidence/credible interval) and measures of statistical heterogeneity. If comparing groups, describe the direction of the effect.	5 & 6
	20c	Present results of all investigations of possible causes of heterogeneity among study results.	5 & 6
	20d	Present results of all sensitivity analyses conducted to assess the robustness of the synthesized results.	Not Applicable
Reporting biases	21	Present assessments of risk of bias due to missing results (arising from reporting biases) for each synthesis assessed.	6
Certainty of evidence	22	Present assessments of certainty (or confidence) in the body of evidence for each outcome assessed.	6
**DISCUSSION**			
Discussion	23a	Provide a general interpretation of the results in the context of other evidence.	6
	23b	Discuss any limitations of the evidence included in the review.	7
	23c	Discuss any limitations of the review processes used.	7
	23d	Discuss implications of the results for practice, policy, and future research.	7
**OTHER INFORMATION**			
Registration and protocol	24a	Provide registration information for the review, including register name and registration number, or state that the review was not registered.	2
	24b	Indicate where the review protocol can be accessed, or state that a protocol was not prepared.	2
	24c	Describe and explain any amendments to information provided at registration or in the protocol.	2
Support	25	Describe sources of financial or non-financial support for the review, and the role of the funders or sponsors in the review.	10
Competing interests	26	Declare any competing interests of review authors.	10 (none declared)
Availability of data, code and other materials	27	Report which of the following are publicly available and where they can be found: template data collection forms; data extracted from included studies; data used for all analyses; analytic code; any other materials used in the review.	7 & supplementary information files

Supplementary Table 7. PRISMA network meta-analysis checklist.

Section/Topic	Item #	Checklist Item	Reported on Page #
**TITLE**			
Title	1	Identify the report as a systematic review *incorporating a network meta-analysis (or related form of meta-analysis).*	1
**ABSTRACT**			
Structured summary	2	Provide a structured summary including, as applicable:<br>**Background:** main objectives<br>**Methods:** data sources; study eligibility criteria, participants, and interventions; study appraisal; and *synthesis methods, such as network meta-analysis.*<br>**Results:** number of studies and participants identified; summary estimates with corresponding confidence/credible intervals; *treatment rankings may also be discussed. Authors may choose to summarize pairwise comparisons against a chosen treatment included in their analyses for brevity.*<br>**Discussion/Conclusions:** limitations; conclusions and implications of findings.<br>**Other:** primary source of funding; systematic review registration number with registry name.	1
**INTRODUCTION**			
Rationale	3	Describe the rationale for the review in the context of what is already known*, including mention of why a network meta-analysis has been conducted.*	2
Objectives	4	Provide an explicit statement of questions being addressed, with reference to participants, interventions, comparisons, outcomes, and study design (PICOS).	2
**METHODS**			
Protocol and registration	5	Indicate whether a review protocol exists and if and where it can be accessed (e.g., Web address); and, if available, provide registration information, including registration number.	2
Eligibility criteria	6	Specify study characteristics (e.g., PICOS, length of follow-up) and report characteristics (e.g., years considered, language, publication status) used as criteria for eligibility, giving rationale. *Clearly describe eligible treatments included in the treatment network, and note whether any have been clustered or merged into the same node (with justification).*	3
Information sources	7	Describe all information sources (e.g., databases with dates of coverage, contact with study authors to identify additional studies) in the search and date last searched.	3

Search	8	Present full electronic search strategy for at least one database, including any limits used, such that it could be repeated.	3
Study selection	9	State the process for selecting studies (i.e., screening, eligibility, included in systematic review, and, if applicable, included in the meta-analysis).	3
Data collection process	10	Describe method of data extraction from reports (e.g., piloted forms, independently, in duplicate) and any processes for obtaining and confirming data from investigators.	3
Data items	11	List and define all variables for which data were sought (e.g., PICOS, funding sources) and any assumptions and simplifications made.	3
**Geometry of the network**	**S1**	Describe methods used to explore the geometry of the treatment network under study and potential biases related to it. This should include how the evidence base has been graphically summarized for presentation, and what characteristics were compiled and used to describe the evidence base to readers.	4
Risk of bias within individual studies	12	Describe methods used for assessing risk of bias of individual studies (including specification of whether this was done at the study or outcome level), and how this information is to be used in any data synthesis.	4
Summary measures	13	State the principal summary measures (e.g., risk ratio, difference in means). *Also describe the use of additional summary measures assessed, such as treatment rankings and surface under the cumulative ranking curve (SUCRA) values, as well as modified approaches used to present summary findings from meta-analyses.*	4
Planned methods of analysis	14	Describe the methods of handling data and combining results of studies for each network meta-analysis. This should include, but not be limited to:<br>• *Handling of multi-arm trials;*<br>• *Selection of variance structure;*<br>• *Selection of prior distributions in Bayesian analyses; and*<br>• *Assessment of model fit.*	4
**Assessment of Inconsistency**	**S2**	Describe the statistical methods used to evaluate the agreement of direct and indirect evidence in the treatment network(s) studied. Describe efforts taken to address its presence when found.	4
Risk of bias across studies	15	Specify any assessment of risk of bias that may affect the cumulative evidence (e.g., publication bias, selective reporting within studies).	4
Additional analyses	16	Describe methods of additional analyses if done, indicating which were pre-specified. This may include, but not be limited to, the following:<br>• Sensitivity or subgroup analyses;<br>• Meta-regression analyses;<br>• *Alternative formulations of the treatment network; and*	4

		• *Use of alternative prior distributions for Bayesian analyses (if applicable).*	
**RESULTS†**			
Study selection	17	Give numbers of studies screened, assessed for eligibility, and included in the review, with reasons for exclusions at each stage, ideally with a flow diagram.	5 & 13
**Presentation of network structure**	**S3**	Provide a network graph of the included studies to enable visualization of the geometry of the treatment network.	5 & 15
**Summary of network geometry**	**S4**	Provide a brief overview of characteristics of the treatment network. This may include commentary on the abundance of trials and randomized patients for the different interventions and pairwise comparisons in the network, gaps of evidence in the treatment network, and potential biases reflected by the network structure.	5
Study characteristics	18	For each study, present characteristics for which data were extracted (e.g., study size, PICOS, follow-up period) and provide the citations.	5 & 13
Risk of bias within studies	19	Present data on risk of bias of each study and, if available, any outcome level assessment.	5 & 14
Results of individual studies	20	For all outcomes considered (benefits or harms), present, for each study: 1) simple summary data for each intervention group, and 2) effect estimates and confidence intervals. *Modified approaches may be needed to deal with information from larger networks.*	5 & 6
Synthesis of results	21	Present results of each meta-analysis done, including confidence/credible intervals. *In larger networks, authors may focus on comparisons versus a particular comparator (e.g. placebo or standard care), with full findings presented in an appendix. League tables and forest plots may be considered to summarize pairwise comparisons.* If additional summary measures were explored (such as treatment rankings), these should also be presented.	5 & 6
**Exploration for inconsistency**	**S5**	Describe results from investigations of inconsistency. This may include such information as measures of model fit to compare consistency and inconsistency models, *P* values from statistical tests, or summary of inconsistency estimates from different parts of the treatment network.	5
Risk of bias across studies	22	Present results of any assessment of risk of bias across studies for the evidence base being studied.	6
Results of additional analyses	23	Give results of additional analyses, if done (e.g., sensitivity or subgroup analyses, meta-regression analyses*, alternative network geometries studied, alternative choice of prior distributions for Bayesian analyses,* and so forth).	6 & 16

**DISCUSSION**			
Summary of evidence	24	Summarize the main findings, including the strength of evidence for each main outcome; consider their relevance to key groups (e.g., healthcare providers, users, and policy-makers).	6 & 7
Limitations	25	Discuss limitations at study and outcome level (e.g., risk of bias), and at review level (e.g., incomplete retrieval of identified research, reporting bias). *Comment on the validity of the assumptions, such as transitivity and consistency. Comment on any concerns regarding network geometry (e.g., avoidance of certain comparisons).*	7
Conclusions	26	Provide a general interpretation of the results in the context of other evidence, and implications for future research.	7
**FUNDING**			
Funding	27	Describe sources of funding for the systematic review and other support (e.g., supply of data); role of funders for the systematic review. This should also include information regarding whether funding has been received from manufacturers of treatments in the network and/or whether some of the authors are content experts with professional conflicts of interest that could affect use of treatments in the network.	10

PICOS = population, intervention, comparators, outcomes, study design.
* Text in italics indicates wording specific to reporting of network meta-analyses that has been added to guidance from the PRISMA statement.
† Authors may wish to plan for use of appendices to present all relevant information in full detail for items in this section.

Supplementary Figure 1. Relative ranking of treatments for the ameloblastoma network based on the multidimensional scaling (MDS) approach.

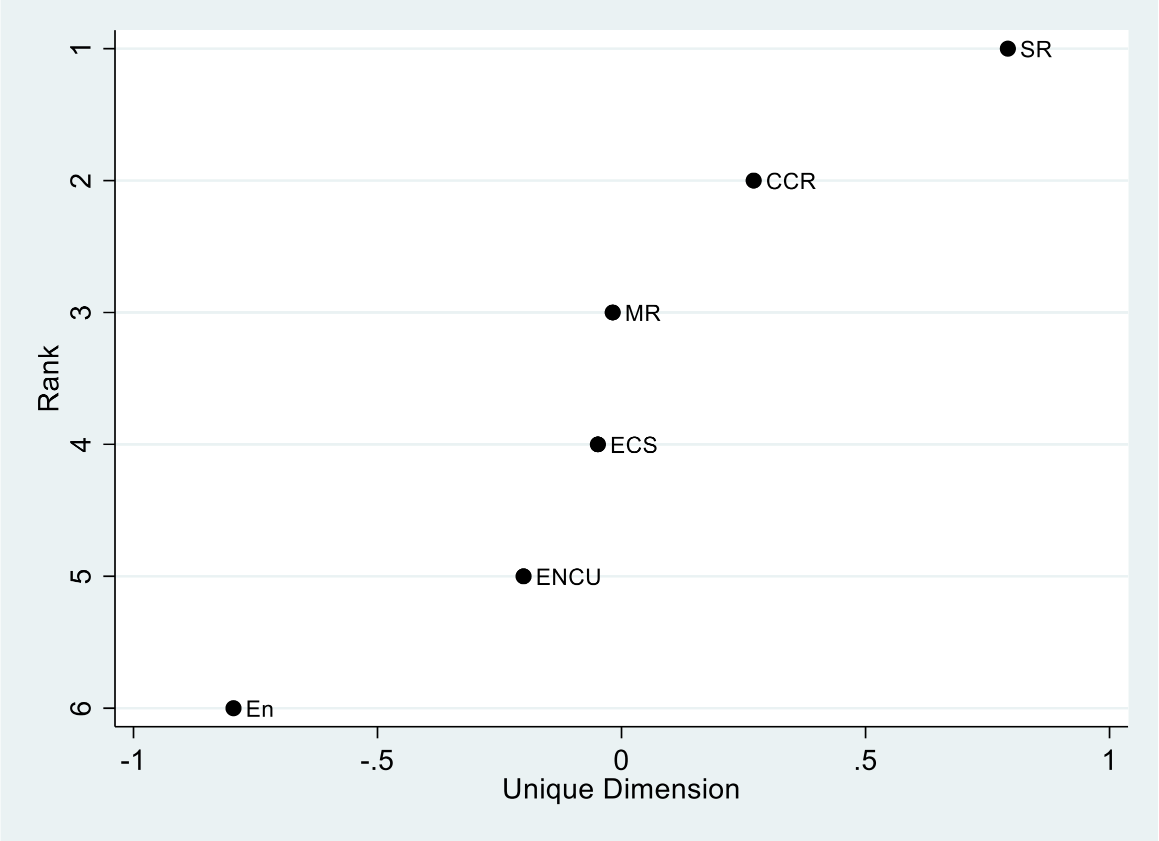

Notes: Larger values of the dimension correspond to higher ranks. CCR = Curettage + Cryotherapy, ECS = Enucleation + Carnoy's solution, En = Enucleation, ENCU = Enucleation + Curettage, MR = Marginal resection, SR = Segmental resection.

Supplementary Figure 2. Comparison-adjusted funnel plot of the ameloblastoma treatments network.

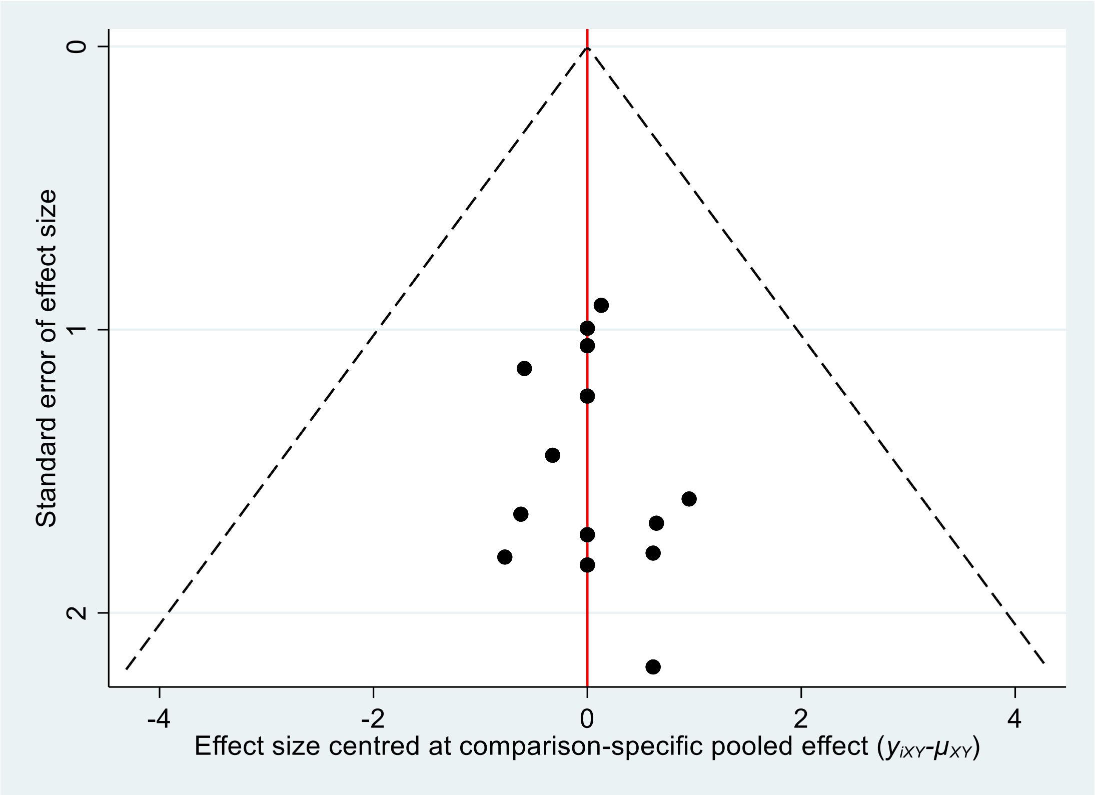

Supplementary Figure 3. Interval plot of treatment approach comparisons for recurrence outcome using Odds Ratio (OR) to measure the effect size.

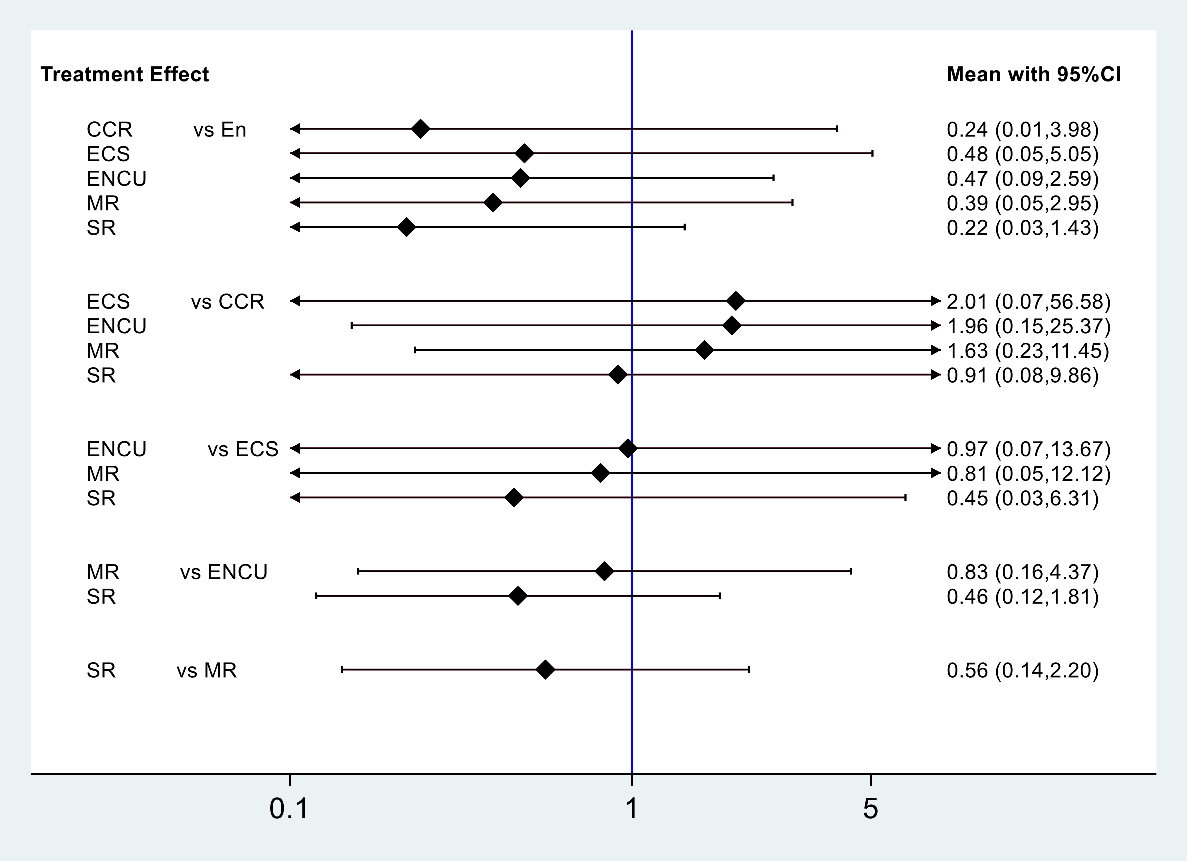

Notes: CCR = Curettage + Cryotherapy, ECS = Enucleation + Carnoy's solution, En = Enucleation, ENCU = Enucleation + Curettage, MR = Marginal resection, SR = Segmental resection.

Supplementary Figure 4. Network forest plots of treatment approach comparisons.

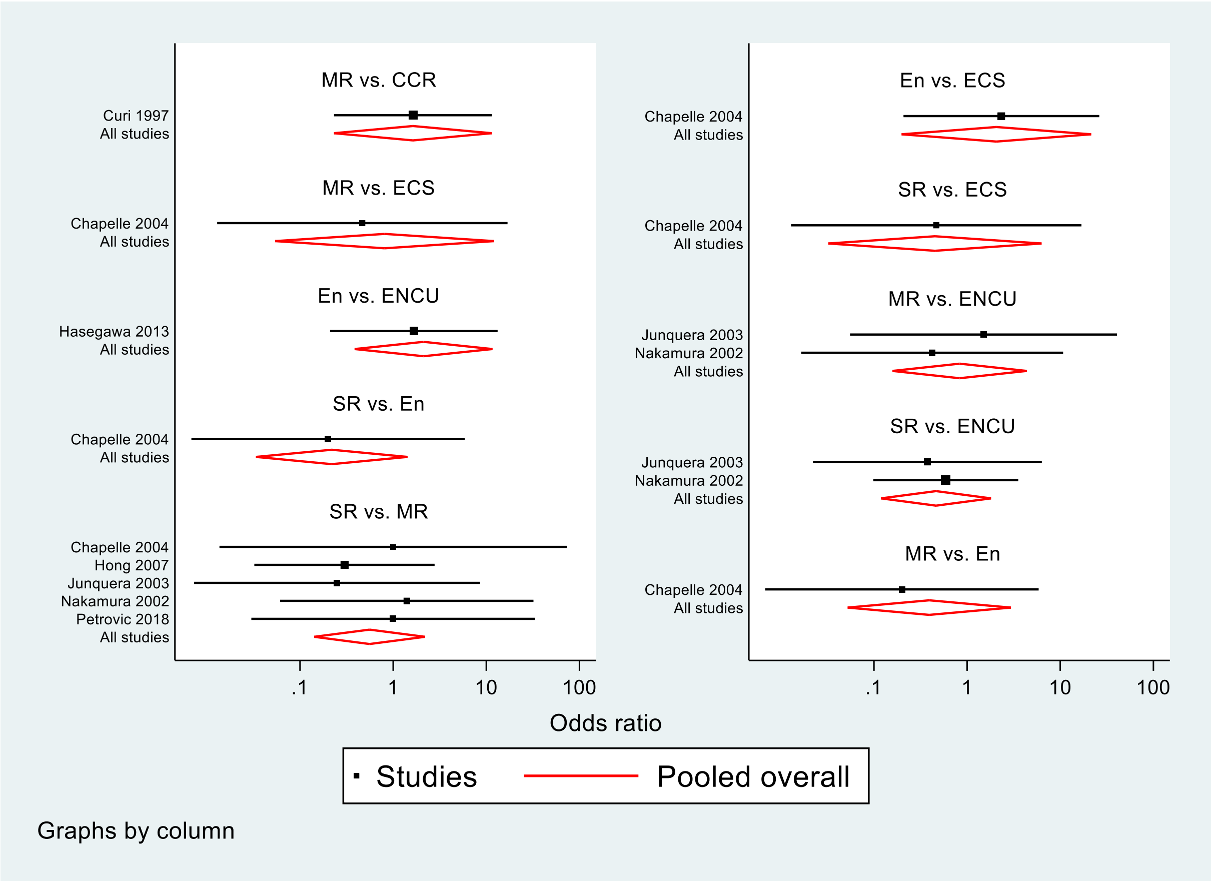

Notes: CCR = Curettage + Cryotherapy, ECS = Enucleation + Carnoy's solution, En = Enucleation, ENCU = Enucleation + Curettage, MR = Marginal resection, SR = Segmental resection.

Supplementary Figure 5. Cumulative probability curves for the ameloblastoma treatments network show that each treatment's estimated and predictive probabilities are up to a specific rank.

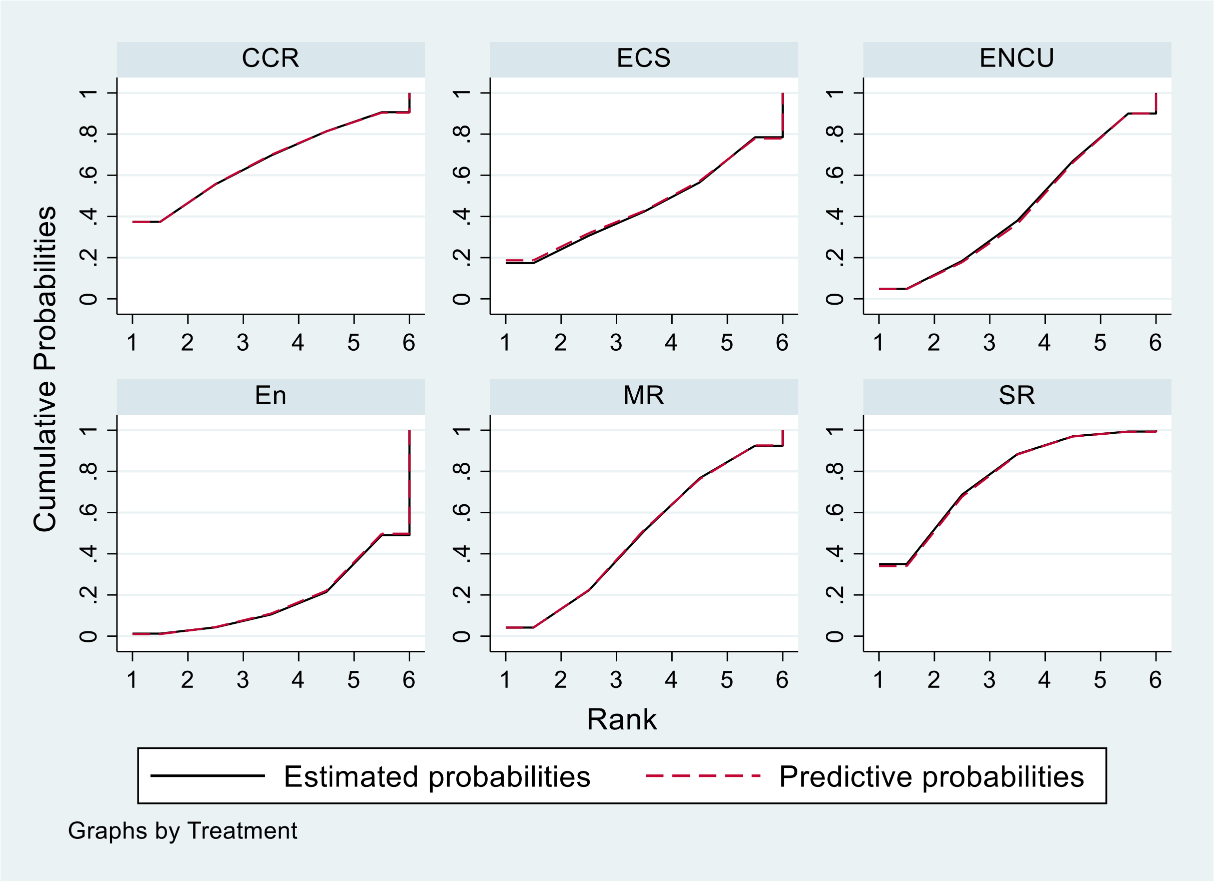

Notes: CCR = Curettage + Cryotherapy, ECS = Enucleation + Carnoy's solution, En = Enucleation, ENCU = Enucleation + Curettage, MR = Marginal resection, SR = Segmental resection.
